# Exploring Factors Associated with Adolescent Tuberculosis in India: Evidence from the National Family Health Survey (2019–21)

**DOI:** 10.3390/diseases14020055

**Published:** 2026-02-02

**Authors:** Ratnakar Singh, Adhin Bhaskar, Jagriti Gupta, Mahalingam Vasantha, Chinnaiyan Ponnuraja

**Affiliations:** Department of Statistics, ICMR-National Institute for Research in Tuberculosis, Chetpet, Chennai 600031, Tamil Nadu, India; sata.ratnakar@icmr.gov.in (R.S.); jagriti.gupta97@icmr.gov.in (J.G.); vasantha.m@icmr.gov.in (M.V.); ponnuraja.c@icmr.gov.in (C.P.)

**Keywords:** tuberculosis, adolescent, associated factors, risk groups, NFHS, generalized linear mixed-effects models (GLMMs)

## Abstract

Background: Tuberculosis (TB) in adolescents is distinct from both childhood and adult TB, particularly in terms of risk factors; however, national-level data assessing these factors in adolescents remain limited despite growing attention to the issue. This study aims to identify factors associated with TB among individuals aged 10 to 18 years. Methods: This study leverages data from the National Family Health Survey (NFHS-5) conducted in India during the year 2019–2021. A total of 479,674 adolescents were included. We employ a generalized linear mixed-effects logistic regression model to examine the association between household, environmental, demographic and behavioral factors and self-reported TB status among adolescents. Results: A total of 363 adolescents reported having TB. The results show that adolescents who are male (aOR = 0.735, *p* < 0.001), living in a nuclear family (aOR = 0.782, *p* < 0.001), residing in a household without TB cases (aOR = 0.17, *p* < 0.001), using a traditional mud stove or chullah (aOR = 0.279, *p* < 0.001), do not have air conditioning or a cooler (aOR = 0.405, *p* < 0.001), do not use tobacco (aOR = 0.766, *p* < 0.001), and do not consume alcohol (aOR = 0.912, *p* < 0.001) have lower odds of TB. Conversely, older age (aOR = 1.136, *p* < 0.001), absence of a separate kitchen (aOR = 1.395, *p* < 0.001), belonging to poor (aOR = 2.787, *p* < 0.005) or middle-income households (aOR = 2.662, *p* < 0.001), and living in households without cattle (aOR = 1.489, *p* < 0.001) are associated with higher odds of TB. Conclusions: Using nationally representative NFHS data, this study identifies multiple household, socioeconomic, and behavioral factors associated with TB among adolescents in India. These findings highlight the need for targeted TB prevention strategies that address household conditions, socio-economic disparities, and adolescent health behaviors.

## 1. Introduction

Tuberculosis (TB) is a centuries-old infectious disease caused by *Mycobacterium tuberculosis (M.TB)*, which has been scientifically studied since Robert Koch’s discovery of the bacillus in 1882 [1]. Globally, TB remains a major public health challenge, with over 10 million incident cases and approximately 1 million deaths each year. India, one of the 30 high TB-burden countries, accounts for about 26% of global TB incidence and mortality [1,2]. With respect to children and adolescents, more than 0.33 million children aged 0–14 years develop TB annually in India. In 2019, 13% of all reported TB cases in India occurred among adolescents, while 6% were pediatric cases [3]. According to the 2011 Census, approximately 253 million individuals in India fall within the 10–19 year age group [2]. As exposure to TB increases with age and the protective effect of the Bacillus Calmette–Guérin (BCG) vaccine wanes, adolescents face a heightened risk of developing TB [4].

TB in adolescents is distinct from both childhood and adult TB, particularly in terms of associated factors and clinical presentation [3]. Adolescents with TB exhibit a combination of features typical of both childhood and adult disease across clinical, radiological, and microbiological parameters [5]. While correlates and risk factors for TB have been extensively studied in adults, they remain relatively underexplored in the adolescent population [6]. Recognizing this gap, the World Health Organization (WHO) issued consolidated guidelines in 2022 to strengthen TB prevention and management in young children and to address unique challenges faced by adolescents [7].

Despite increasing attention on adolescent TB, national-level data assessing factors associated with TB in this age group remain limited. Therefore, in this study, we aim to leverage data from the National Family Health Survey (NFHS-5) to identify factors associated with TB among individuals aged 10 to 18 years in India. Given that NFHS primarily collects household- and individual-level survey data rather than clinical diagnostic information, we examine how household, sociodemographic, and environmental characteristics are associated with self-reported TB among adolescents.

## 2. Materials and Methods

### 2.1. Data Sources and Participants

This study used data from the NFHS-5, conducted in India between 2019 and 2021. The survey achieved high participation rates, with a household response rate of 98%, and individual response rates of 97% for women and 92% for men, ensuring strong representativeness of the survey population [8]. NFHS is a large-scale, multi-round survey administrated to a nationally representative sample of households, providing extensive health and demographic information across India’s administrative hierarchy, including states, districts, residence type (urban/rural), clusters, households, and individuals. Since its first round in 1992–1993, each NFHS round has aimed: (a) generate essential national and state-level data on health and family welfare, and (b) provide insights into emerging population health issues. It is important to note that NFHS-5 is not specifically designed to investigate TB or its associated factors. TB information is limited to a self-reported item; therefore, the present study constitutes a secondary, exploratory analysis using the available survey variables. NFHS-5 employs a multistage stratified sampling design. States and Union Territories (UTs) are stratified into urban and rural areas [8]. Clusters (census enumeration blocks in urban areas and villages in rural areas) are selected with probability proportional to size, followed by random selection of households within each cluster. All eligible individuals within selected households are interviewed.

The sampling framework ensures representativeness of the adolescent population across India’s diverse regions. NFHS-5 surveyed 2,843,917 individuals in total. For this study, we restricted the dataset to adolescents aged 10–18 years, yielding a final analytical sample of 479,674 individuals. Information on TB history and related variables was obtained through standardized questionnaires. In NFHS-5, individual interviews were conducted only for women aged 15–49 years and men aged 15–54 years [8]. Consequently, for adolescents aged 10–14 years, TB-related and other health information were reported by the household respondent or primary caregiver. For adolescents aged 15–18 years, information was self-reported when the adolescent was selected for an individual interview. Thus, depending on age, TB-related responses originate either directly from the adolescent or indirectly via an adult household respondent. Variables included in the TB modeling span a wide range of sociodemographic, environmental, and health-related characteristics, such as household cooking fuel, presence of separate kitchen, ventilation, toilets sharing, livestock ownership, wealth index, smoking and tobacco use within households, alcohol consumption, anemia status, body mass index (BMI), and self-reported TB among household members. TB status in NFHS-5 wa based on self-report and did not include microbiological, radiological, or clinical confirmation. Therefore, the outcome used in this study reflects self-reported TB status rather than confirmed TB diagnosis.

### 2.2. Data Processing and Statistical Analysis

The primary outcome was self-reported TB status of the adolescents, based on the NFHS-5 question: “Are you suffering from TB?” This variable was coded as binary (presence or absence of TB). All independent variables were categorical, with age being the only continuous predictor. Data cleaning and statistical analyses are performed in R software (version 4.5.1). Missing data were handled using complete-case analysis; records missing the outcome (self-reported TB) were excluded. Missingness among independent variables is assessed, and variables with more than 10% missing data were omitted from adjusted analyses. Because NFHS-5 used a complex, multistage survey design, and the data have a hierarchical structure (individuals nested within households, clusters, residence type, districts and states), we employed nested generalized linear mixed-effects models (GLMMs) to account for clustering at multiple levels. GLMMs with a binomial family and logit link were used to examine associations between TB status and various household, environmental, demographic and behavioral factors.

Unadjusted logistic regression analyses were first conducted to assess association between each predictor and TB status. Variables with *p*-values < 0.20 in these unadjusted analyses were included in the multivariable model. Multicollinearity was assessed using variance inflation factor and found to be within acceptable limits.

GLMMs extend generalized linear models by incorporating both fixed and random effects [9]. They are particularly appropriate for hierarchical or clustered data, where observations within the same group (e.g., household or cluster) may be correlated due to shared environmental and socioeconomic conditions. Random intercepts are included to account for this intra-group correlation and to produce more reliable parameter estimates.

The binary outcome variable $Y_{ihcrds}$ represents the TB status (1 = TB present, 0 = TB absent) for individual i in household h, cluster c, residence type r, district d, and state s. The model accounts for the hierarchical and nested structure of the data by including random intercepts at multiple levels (1):

(1) $$logit\left(P\left(Y_{ihcrds}=1\right)\right)=\beta_{0}+\sum_{m=1}^{M}\beta_{m}X_{ihcrds}^{m}+uₛ+u_{d}(s)+u_{r}(d,s)+u_{c}(r,d,s)+u_{h}(c,r,d,s)+u_{i}\left(h,c,r,d,s\right)$$

where

- $Y_{ihcrds}$ represents an individual i nested within household h, cluster c, residence type r, district d, and state s, reflecting the multilevel structure of the NFHS-5 data.
- $\beta_{0}$ is the fixed intercept.
- $\beta_{m}$ are fixed-effect coefficients for predictor variables $X^{m}$ including sleep room sharing, separate kitchen, wealth index, cooking method, livestock ownership, sex, indoor smoking, air conditioner/cooler use, kitchen ventilation, tobacco use, alcohol consumption, household structure, age, and presence of TB in the household.
- $u_{s},u_{d},u_{r},u_{c},u_{h},u_{i}$ are random intercepts for state s, district d, residence type r, cluster c, household h, and individual i, respectively, capturing unobserved heterogeneity at each nested level.

In the present study, the binary outcome was self-reported TB status of adolescents. The GLMM was used to examine the relationship between this outcome and various explanatory variables, including household conditions, demographic characteristics, and behavioral factors. In a mixed-effects framework, fixed effects estimate the average population-level associations between predictors and TB status, while random effects account for unobserved variability across different hierarchical levels. For this analysis, we treated six levels as grouping factors: State, District, Residence Type (urban/rural), Clusters, Household, and Individual. Random intercepts were specified at each level to appropriately model the nested structure of the NFHS-5 data and to adjust for intra-group correlation. These random effects capture unmeasured variation at each level, such as differences in TB risk across states or between households, beyond what is explained by the fixed covariates. A GLMM with a binomial distribution and logit link function was used to model the probability of reporting TB. This approach allowed us to estimate the log-odds of TB as a function of multiple individual and household-level predictors while accounting for residual variance attributed to the nested data structure. All analyses using GLMMs were implemented through the *glmer()* function from the *lme4* package in the R programming environment [10]. Final model results were reported as adjusted odds ratios (aORs) with 95% confidence intervals (CIs) and statistical significance is set at *p* < 0.05. As this study did not attempt causal inference, these estimates should be interpreted as associations rather than causal effect relationships, thereby avoiding the “Table 2 fallacy” [11].

## 3. Results

### 3.1. Basic Demographics

A total of 479,674 adolescents aged 10–18 years were included in the analyses. Among them, 363 adolescents (0.08%) self-reported having TB, of whom 173 (47.66%) were male. The mean age of adolescents with TB was 14.84 years (SD = 2.62). Among those reporting TB, 107 (38.35%) lived in households without separate kitchen. By wealth status, 214 (58.95%) belonged to poor households, 71 (19.56%) to middle-income households, and 78 (21.49%) to rich households. In terms of household structure, 231 (63.64%) lived in nuclear families and 44 (12.12%) reported the presence of another TB case within the household. By BMI category, 49 (44.14%) were underweight, 56 (50.45%) had normal BMI, and 6 (5.41%) were overweight.

Regarding cooking methods, 155 (76.00%) reported that food in their household was cooked using a chullah, 43 (21.00%) using an open fire, and 5 (2.00%) using a stove. A total of 104 (28.65%) lived in households with cattle (cows, bulls, buffaloes, or yaks), and 20 (9.8%) reported sharing sleeping space with animals. Household smoking exposure was high, with 200 adolescents (55.1%) living in homes where someone smoked indoors. Only 38 (10.47%) had access to air conditioning or a cooler, and 221 (79.21%) reported the presence of ventilation in the kitchen. Regarding personal behaviors, 17 adolescents (7.94%) reported tobacco use, 7 (3.26%) reported consuming alcohol, and 59 (55.66%) were anemic.

A detailed distribution of socio-demographic and household characteristics among adolescents with self-reported TB, along with unadjusted odds ratios (ORs), 95% confidence intervals (CIs), and *p*-values, is provided in Table 1.

### 3.2. Geographic Distribution of Adolescents TB Cases in India

**Figure 1:**
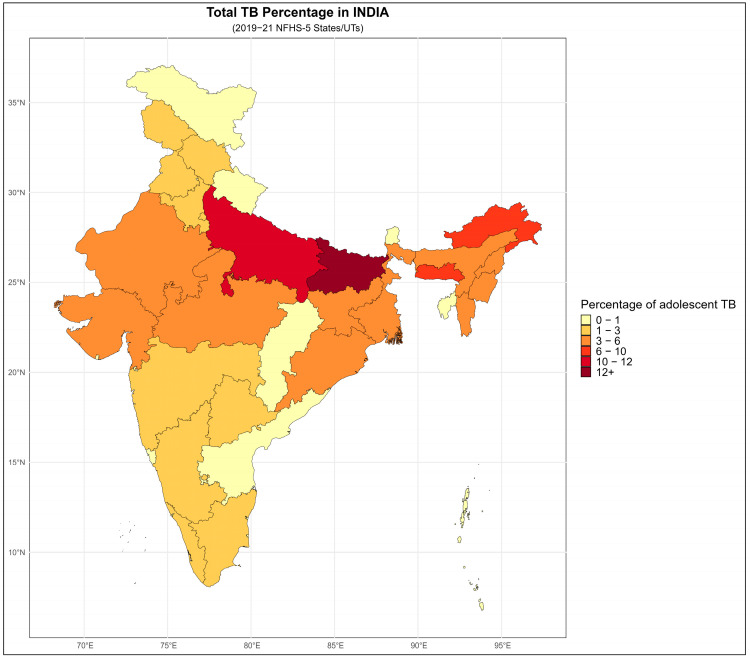
Distribution of adolescents TB cases across different States/UTs of India, NFHS-5 (2019–21).

The geographic distribution of the 363 adolescents with self-reported TB in NFHS-5 showed considerable regional variation across India (Figure 1): Bihar (13.77%) reported the highest proportion of adolescent TB cases, followed by Uttar Pradesh (11.57%), Meghalaya (8.82%) Arunachal Pradesh (6.34%) and Nagaland (4.96%), which collectively represented the states with the highest reported burden. Moderate proportions of cases were observed in Assam (4.41%), Gujarat (4.13%), Manipur (4.13%), West Bengal (3.58%), and a cluster of states–including Jharkhand, Madhya Pradesh, Mizoram, and Rajasthan–each contributing approximately 3.31% of the total cases. Odisha (3.03%) and Karnataka (2.75%) also fall within this intermediate range. Several large states-including Tamil Nadu, Telangana, Kerala, Delhi, and Punjab-report relatively lower proportions, ranging from 1.1% and 2.48%. Similarly, Haryana, Himachal Pradesh, Maharashtra, and Jammu and Kashmir also fall into lower-burden category. A group of Union Territories and smaller states such as Andaman and Nicobar Islands, Chandigarh, Sikkim, Tripura, Chhattisgarh, Lakshadweep, and Uttarakhand reported very low proportions (≤ 0.83%). Goa, Puducherry, Ladakh, and Dadra and Nagar Haveli reported zero TB cases of adolescents.

### 3.3. Unadjusted Analysis

The unadjusted analysis showed that several household-level and individual level factors were significantly associated with self-reported TB. Adolescents from nuclear households had significantly higher odds of TB compared to those from non-nuclear households (OR = 1.267, 95% CI (1.023–1.569), *p* < 0.05). Living in a household with a separate kitchen was associated with reduced odds of TB (OR = 0.785, 95% CI (0.616–0.999), *p* < 0.05). Similarly, the presence of kitchen ventilation was associated with lower odds of TB (OR = 0.725, 95% CI (0.543–0.968), *p* < 0.05).

Adolescents living in homes where food was cooked on a chullah were significantly less prone to TB (OR = 0.278, 95% CI (0.198–0.390), *p* < 0.001). Those belonging to poor households had a significantly higher likelihood of TB compared with adolescents from rich households (OR = 1.615, 95% CI (1.246–2.093), *p* < 0.001).

Individual behaviors were also associated with TB. Adolescent who smoked or used tobacco (OR = 2.042, 95% CI (1.244–3.354), *p* < 0.005) or consumed alcohol (OR = 2.546, 95% CI (1.198–5.411), *p* < 0.05) had significantly higher odds of TB. Having a usual household member with TB was strongly associated with increased odds of TB in adolescents (OR = 12.388, 95% CI (9.028–16.998), *p* < 0.001). Belonging to households that owned cows, buffaloes, or yaks was associated with lower odds of TB (OR = 0.482, 95% CI (0.384–0.605), *p* < 0.001). BMI category was not significantly associated with TB, although underweight showed a non-significant trend toward higher TB odds (OR = 1.285, CI (0.875–1.886), *p* = 0.200).

### 3.4. GLMM

For the multi-variable mixed effect model, Type I analyses were performed to identify variables with *p*-value ≤ 0.20, which were then included in the multi-variable GLMM. The GLMM with nested random intercepts at the state, district, residence type, household, and individual levels was statistically significant, indicating meaningful variation in TB across all hierarchical levels.

The results of the GLMM assessing socio-demographic and household characteristics associated with self-reported TB among adolescents are presented in Table 2, along with the adjusted odds ratios (aORs), 95% CIs, and corresponding *p*-values (Table 2). These model estimates reflect associations, not causal effect relationships. Several household-level and individual level factors were significantly associated with self-reported TB. Significant predictors included presence of a separate kitchen room, household wealth status, cooking methods, livestock ownership (cows, bulls, buffaloes, or yaks), gender, availability of air conditioning or cooler, self-reported tobacco use, alcohol consumption, household structure, age and, having a usual household member with TB. Male adolescents had significantly lower odds of TB (aOR = 0.735, 95% CI (0.733–0.736), *p* < 0.001) compared to female adolescents. Increasing age was associated with higher odds of TB (aOR = 1.136, 95% CI (1.134–1.138), *p* < 0.001).

Adolescents living in a nuclear households had lower odds of TB (aOR = 0.782, 95% CI (0.780–0.783), *p* < 0.001) compared to those in non-nuclear households. Living in a household without any usual resident who had TB was strongly protective (aOR = 0.17, 95% CI (0.17–0.17), *p* < 0.001). Adolescents from households without a separate kitchen had significantly higher odds of TB (aOR= 1.395, 95% CI (1.392–1.398), *p* < 0.001).

Regarding cooking method, adolescents from households using a chullah had substantially lower odds of TB (aOR = 0.279, 95% CI (0.278–0.279), *p* < 0.001) compared to those using open fire. Not having an air conditioning or cooler was associated with reduced odds of TB (aOR = 0.405, 95% CI (0.404–0.406), *p* < 0.001). Wealth status showed a clear gradient. Adolescents from poor households had markedly higher odds to TB (aOR = 2.787, 95% CI (1.445–5.377), *p* < 0.005) compared to those from rich households, while adolescents from middle-income households also showed elevated odds (aOR = 2.662, 95% CI (2.657–2.667), *p* < 0.001). Adolescents living in households without livestock had higher odds of TB (aOR = 1.489, 95% CI (1.486–1.492), *p* < 0.001). Those who did not smoke or use tobacco (aOR = 0.766, 95% CI (0.765–0.768), *p* < 0.001) and those who did not consume alcohol (aOR = 0.912, 95% CI (0.910–0.914), *p* < 0.001) had significantly lower odds of TB compared to adolescents who engaged in these behaviors.

## 4. Discussion

To the best of our knowledge, this is the first study to examine socio-demographic, environmental, and behavioral factors associated with TB among adolescents using a nationally representative dataset from a lower-middle-income country. Adolescent TB in India exhibits a complex epidemiological pattern, with prevalence estimates varying widely across studies due to differences in design, setting, and measurement approaches. In India, approximately 0.12% of the adolescent population were notified with TB in 2019, indicating that although adolescents account for a relatively small proportion of reported cases, the true burden is likely higher because of under-diagnosis and under-notification in this age group [3]. Bhargava et al. (2020) reported 377 TB cases among 277,059 adolescents, identifying poverty and urban residence as key determinants [12]. Active surveillance data from Uppada et al. (2016) estimated an incidence of 147.6 per 100,000 person-years [13]. The comparatively lower number of cases observed in our NFHS-5 analysis was consistent with the use of a single self-reported household question, which inherently underestimates the true TB burden due to undiagnosed disease and stigma-related non-disclosure.

Several important findings emerged from this study. A key observation was that male adolescents had significantly lower odds of TB compared with females. This aligns with findings from India by Thakur et al. (2021), who reported higher vulnerability among adolescent females, possibly due to biological factors, undernutrition, and sociocultural restrictions that limit healthcare access for females [14]. Age also emerged as an important correlate, with older adolescents having higher odds of TB. Similar trends have been noted in Ethiopia, where Dememew et al. (2025) observed a higher burden of TB among older female adolescents [15]. Increasing age may correspond to greater exposure to environmental and behavioral risk factors. Additionally, evidence suggests that the protective effect of BCG vaccine wanes after approximately 10 years, which may contribute to increasing susceptibility to TB during adolescence [4,16]. The waning immunity highlights the potential value of adolescent-targeted vaccines or BCG revaccination strategies [17].

Household and family structure played a notable role. Although the unadjusted analysis initially indicated higher odds of TB in nuclear families, this relationship reversed in the multivariable GLMM after adjusting for multiple confounders. The unadjusted association was likely influenced by socioeconomic status, crowding, and household TB exposure. After adjustments, nuclear families were associated with lower odds of TB, potentially due to reduced household density, fewer interpersonal contacts, and lower risk of intra-household transmission. Consistent with this interpretation, the absence of any TB case among household members was strongly protective, supporting earlier findings from China, which emphasized household exposure as a dominant transmission pathway [18,19].

Housing and cooking conditions were also strongly associated with TB. Adolescents from households without a separate kitchen had significantly higher odds of TB, consistent with studies from Bangladesh and India demonstrating the role of indoor air pollution and poor ventilation in increasing TB risk [18,20]. Notably, adolescents from households using a chullah had lower odds of TB than those using open fires, suggesting that more controlled cooking environments may mitigate exposure to harmful smoke or pathogens [21]. Interestingly, the lack of air conditioning or cooling devices was associated with lower TB odds. Although existing research does not directly address cooling devices, prior research highlights the importance of ventilation and household crowding in TB transmission [22,23]. Further investigation is needed to better understand the mechanism underlying this association.

In this study, socioeconomic status, measured through the wealth index, was a strong predictor of TB. Adolescents from poor and middle-income households had significantly higher odds of TB than those from rich households. This finding were consistent with the established inverse relationship between socioeconomic development and TB incidence, often mediated by malnutrition, healthcare access, and living conditions. Numerous studies have demonstrated that lower socio-demographic index regions carry disproportionately higher TB burden [24,25,26,27,28,29].

Another important observation was the role of livestock ownership in the household. The multivariable analysis showed that adolescents living in households without cattle had significantly higher odds of developing TB. This association may be due to the fact that households owning livestock often have a better standard of living and improved nutritional intake. However, the potential role of bovine TB transmission should also be considered. Close contact with infected cattle or consumption of unpasteurized dairy products may increase the risk of zoonotic Mycobacterium bovis infection [30,31].

Behavioral factors such as tobacco and alcohol use were strongly associated with TB. Adolescents who did not use tobacco or alcohol had significantly lower odds of TB, affirming the findings of previous global studies that linked these substances to increased TB susceptibility due to immune suppression and lung damage [32,33,34].

Previous analyses using NFHS-4 household data have described self-reported TB patterns in the overall Indian population and highlighted strong socio-economic and environmental gradients. Singh et al. used NFHS-4 to show age, sex, educational attainment, marital status, place of residence, wealth index were strongly associated with higher self-reported TB, while Mazumdar et al. reported higher self-reported TB prevalence among socio-economically disadvantaged and less educated groups [35,36]. Our findings in adolescents, higher odds of TB among those from poorer households and in homes without a separate kitchen or with suboptimal household conditions are broadly consistent with these earlier NFHS-4 observations. A more recent analysis comparing NFHS-4 and NFHS-5 reported changes in self-reported TB prevalence and TB knowledge in the general population, including improvements in correct TB knowledge but persistence of misconceptions and stigma [37]. By focusing specifically on adolescents in NFHS-5, our study complements this work by identifying adolescent-specific socio-demographic, environmental, and behavioral correlates of self-reported TB, within the broader pattern of TB burden documented in NFHS-based analyses. Also, previous NFHS-based analyses have highlighted marked geographic heterogeneity in self-reported TB prevalence in India. Using NFHS-4 and NFHS-5 data from the Andaman and Nicobar Islands, Thiruvengadam et al. reported a substantial decline in self-reported TB between the two rounds, but with a consistently higher burden among rural and poorer subpopulations and particularly elevated prevalence in the Nicobar district [38]. Similarly, Muniyandi et al. showed that in Tamil Nadu, district-level self-reported TB prevalence varied widely and that TB cases were spatially clustered, with identifiable hotspot districts across both NFHS-4 and NFHS-5 [39]. In line with these findings, the present study also demonstrates prominent regional variability in adolescent TB, with a concentration of cases in certain states such as Bihar, Uttar Pradesh, and northeastern states. The convergence of our adolescent NFHS-5 results with these NFHS-based spatial analyses suggests that adolescent TB, like all-age TB, is geographically clustered, and that high-burden states or districts would benefit from targeted adolescent-focused screening and prevention strategies.

While earlier NFHS-4 and NFHS-5 studies primarily focused on all-age or adult self-reported TB and its spatial patterns, our study contributed by isolating adolescents and examining how socio-demographic and household characteristics shape their TB risk within the broader geographic heterogeneity of India. By restricting the analysis to adolescents within the NFHS-5 sample and applying a multilevel GLMM framework, we provided age-specific associated factors that were not previously quantified in NFHS-based TB research.

## 5. Limitations

While this study offers valuable insights into adolescent TB at a national level, some limitations must be considered when interpreting the findings of this study. First, the prevalence of self-reported TB among adolescents in the NFHS-5 was very low. Although this limits statistical power and results in wider confidence intervals, the outcome is itself epidemiologically meaningful and reinforces the importance of large, population-based datasets such as NFHS-5 for studying low-frequency conditions. The use of multilevel generalized linear mixed-effects models helped mitigate precision loss by appropriately accounting for the hierarchical structure of the survey data. Second, TB status in NFHS-5 is based on self-report rather than microbiological or radiological confirmation. This introduces the possibility of reporting bias, misclassification, particularly in settings where TB-related stigma may discourage disclosure. Nonetheless, self-reported TB has been widely used in previous national analyses, remains the only TB-related indicator available in NFHS, and offers an important starting point for identifying household-level correlates in a population where diagnostic data are not routinely collected. Third, the cross-sectional design of NFHS-5 does not allow determination of temporal or causal effect relationships. Therefore, the associations identified here should be interpreted as exploratory correlates. However, the goal of the present study was not causal inference but to characterize patterns of association in a nationally representative adolescent population—a purpose for which cross-sectional data remain highly informative. Fourth, complete-case analysis and exclusion of variables with substantial missingness may introduce some degree of selection or omitted-variable bias. Nevertheless, the overall proportion of missing data was small, and careful variable screening ensured that the final model included conceptually relevant and statistically stable predictors. Finally, NFHS-5 did not collect clinical details such as symptomatology, TB subtype, treatment history, or timing of disease onset. Although these limitations restrict the clinical granularity of the model, the survey’s extensive sociodemographic and household-level information enables a broad, population-level assessment of structural and environmental correlates of TB—an area where evidence for adolescents has been notably scarce. Despite these constraints, the present analysis leverages one of the largest and most representative datasets available in India, applies a rigorous multilevel modeling framework, and provides novel, nationally relevant insights into the socio-demographic and household factors associated with adolescent TB. These findings can serve as a foundation for more detailed clinical or longitudinal studies and help guide targeted public health strategies.

## 6. Conclusions

This study provides one of the first national-level assessments of factors associated with self-reported TB among adolescents in India using the NFHS-5 dataset. By applying a rigorous multilevel statistical framework to nearly half a million adolescents, we were able to identify and quantify key socio-demographic, household, and behavioral correlates of TB in this age group. The findings highlighted the role of structural determinants—such as household composition, cooking environment, wealth status, and the presence of TB within the household—alongside individual behaviors that may influence susceptibility. Despite the inherent limitations of self-reported data, the study demonstrated the substantial value of large-scale population surveys for understanding TB patterns in groups that remain underrepresented in epidemiological research. The clear and consistent associations observed across multiple domains underscore the need for adolescent-specific strategies in India’s TB response. Programmatically, our findings support the integration of adolescent-focused TB screening within school health platforms, the strengthening of household-level interventions to reduce environmental exposures, and targeted outreach to socio-economically disadvantaged groups. These measures could complement ongoing national TB elimination efforts by addressing vulnerabilities unique to adolescents. This study contributes important evidence toward shaping public health policies and research priorities, and underscores the urgent need for dedicated adolescent TB surveillance and prevention strategies in India.

## Figures and Tables

**Table 1:** Unadjusted analyses and distribution of socio-demographic and household characteristics among Adolescents with self-reported Tuberculosis (TB) status.

Variables	Suffers from TB		Total N (%)	OR (95% CI)	*p*-Value
	Yes n (%)	No n (%)			
**Age (years)**	14.84 (2.62) *	13.99 (2.61) *	14.00 (2.62) *	1.135 (1.090, 1.182)	<0.001
**Sex of household**					
Male	173 (47.66)	245,232 (51.17)	245,405 (51.16)	0.869 (0.707, 1.068)	0.182
Female	190 (52.34)	234,044 (48.83)	234,234 (48.83)		
**Cooking method (In this household, is food cooked on a stove, a chullah (traditional mud stove) or an open fire?)**					
Stove	5 (2.00)	2007 (1.00)	2012 (1.00)	1.068 (0.423, 2.700)	0.889
Chullah	155 (76.00)	238,964 (92.00)	239,119 (92.00)	0.278 (0.198, 0.390)	<0.001
Open fire	43 (21.00)	18,351 (7.00)	18,394 (7.00)		
**Household has separate room used as kitchen**					
Yes	172 (61.65)	249,594 (67.2)	249,766 (67.2)	0.785 (0.616, 0.999)	<0.05
No	107 (38.35)	121,827 (32.8)	121,934 (32.8)		
**Does the room used as kitchen have any ventilation?**					
Yes	221 (79.21)	312,063 (84.02)	312,284 (84.02)	0.725 (0.543, 0.968)	<0.05
No	58 (20.79)	59,358 (15.98)	59,416 (15.98)		
**Household members smoke inside the house**					
Yes	200 (55.10)	244,324 (50.97)	244,524 (50.98)	1.18 (0.96, 1.451)	0.117
No	163 (44.90)	234,984 (49.03)	235,147 (49.02)		
**Air conditioner/cooler**					
Yes	38 (10.47)	96,979 (20.23)	97,017 (20.23)	0.461 (0.329, 0.645)	<0.001
No	325 (89.53)	382,332 (79.77)	382,657 (79.77)		
**Household structure**					
Nuclear	231 (63.64)	278,038 (58.01)	278,269 (58.01)	1.267 (1.023, 1.569)	<0.05
Non-nuclear	132 (36.36)	201,273 (41.99)	201,405 (41.99)		
**Wealth index combined**					
Poor	214 (58.95)	241,425 (50.37)	241,639 (50.38)	1.615 (1.246, 2.093)	<0.001
Middle	71 (19.56)	95,801 (19.99)	95,872 (19.99)	1.350 (0.979, 1.862)	0.067
Rich	78 (21.49)	142,085 (29.64)	142,163 (29.64)		
**Owns Cows/bulls/buffaloes/yaks**					
Yes	104 (28.65)	217,941 (45.47)	218,045 (45.46)	0.482 (0.384, 0.605)	<0.001
No	259 (71.35)	261,370 (54.53)	261,629 (54.54)		
**Does this household share any sleeping room with this (these) animal(s)?**					
Yes	20 (9.80)	25,855 (9.00)	25,875 (9.00)	1.099 (0.693, 1.744)	0.688
No	184 (90.20)	261,496 (91.00)	261,680 (91.00)		
**Smokes or uses tobacco**					
Yes	17 (7.94)	8642 (4.05)	8659 (4.06)	2.042 (1.244, 3.354)	<0.005
No	197 (92.06)	204,533 (95.95)	204,730 (95.94)		
**Drinks alcohol**					
Yes	7 (3.26)	2782 (1.30)	2789 (1.31)	2.546 (1.198, 5.411)	<0.05
No	208 (96.74)	210,435 (98.7)	210,643 (98.69)		
**Anemia level**					
Anemic	59 (55.66)	56,757 (58.84)	56,816 (58.84)	0.878 (0.599, 1.289)	0.507
Not Anemic	47 (44.34)	39,705 (41.16)	39,752 (41.16)		
**Body Mass Index**					
Underweight	49 (44.14)	37,693 (35.50)	37,742 (35.51)	1.285 (0.875, 1.886)	0.200
Overweight	6 (5.41)	13,133 (12.37)	13,139 (12.36)	0.452 (0.195, 1.048)	0.064
Normal	56 (50.45)	55,347 (52.13)	55,403 (52.13)		
**Does any usual resident of your household suffer from tuberculosis?**					
Yes	44 (12.12)	5278 (1.10)	5322 (1.11)	12.388 (9.028, 16.998)	<0.001
No	319 (87.88)	474,033 (98.90)	474,352 (98.89)		

* Mean (Standard Deviation).

**Table 2:** Generalized Linear Mixed Model (GLMM) analysis of socio-demographic and household characteristics associated with self-reported Tuberculosis among Adolescents.

Variable	Estimate	Std Error	z Value	aOR (95%CI)	*p* Value
**Age (years)**	0.127	0.001	142.458	1.136 (1.134, 1.138)	<0.001
**Sex of household**					
Male	−0.308	0.001	−347.11	0.735 (0.733, 0.736)	<0.001
Female	Reference				
**Cooking method (In this household, is food cooked on a stove, a chullah (traditional mud stove) or an open fire?)**					
Stove	0.891	0.944	0.944	2.438 (0.383, 15.507)	0.345
Chullah	−1.277	0.001	−1295.99	0.279 (0.278, 0.279)	<0.001
Open Fire	Reference				
**Household has separate room used as kitchen**					
No	0.333	0.001	337.986	1.395 (1.392, 1.398)	<0.001
Yes	Reference				
**Does the room used as kitchen have any ventilation?**					
No	0.037	0.457	0.082	1.038 (0.424, 2.54)	0.935
Yes	Reference				
**Household members smoke inside the house**					
No	0.052	0.367	0.14	1.053 (0.513, 2.163)	0.888
Yes	Reference				
**Air conditioner/cooler**					
No	−0.904	0.001	−917.612	0.405 (0.404,0.406)	<0.001
Yes	Reference				
**Household structure**					
Nuclear	−0.246	0.001	−249.722	0.782 (0.78, 0.783)	<0.001
Non-nuclear	Reference				
**Wealth Index**					
Poor	1.025	0.335	3.058	2.787 (1.445, 5.377)	<0.005
Middle	0.979	0.001	993.784	2.662 (2.657, 2.667)	<0.001
Rich	Reference				
**Owns Cows/bulls/buffaloes/yaks**					
No	0.398	0.001	403.855	1.489 (1.486, 1.492)	<0.001
Yes	Reference				
**Smokes or uses tobacco**					
No	−0.266	0.001	−270.483	0.766 (0.765, 0.768)	<0.001
Yes	Reference				
**Drinks alcohol**					
No	−0.092	0.001	−93.753	0.912 (0.91, 0.914)	<0.001
Yes	Reference				
**Does any usual resident of your household suffer from tuberculosis?**					
No	−1.773	10^−8^	−766,923	0.17 (0.17, 0.17)	<0.001
Yes	Reference				

## Data Availability

The data supporting the findings of this study are available from the DHS Program (https://dhsprogram.com/Data/terms-of-use.cfm (accessed on 11 July 2025)). However, due to licensing restrictions, these data are not publicly accessible. Access to the data may be obtained from the authors upon reasonable request and with permission from the DHS Program.

## Abbreviations

The following abbreviations are used in this manuscript:

TB	Tuberculosis
M.TB	Mycobacterium Tuberculosis
BCG	Bacillus Calmette–Guérin
WHO	World Health Organization
NFHS	National Family Health Survey
BMI	Body Mass Index
SD	Standard deviations
Min	Minimum
Max	Maximum
GLMM	Generalized Linear Mixed-Effects Model
OR	Odds Ratio
CI	Confidence Interval
aOR	Adjusted Odds Ratio
UTs	Union Territories
